# Meroterpenes from Marine Invertebrates: Structures, Occurrence, and Ecological Implications

**DOI:** 10.3390/md11051602

**Published:** 2013-05-17

**Authors:** Marialuisa Menna, Concetta Imperatore, Filomena D’Aniello, Anna Aiello

**Affiliations:** The NeaNat Group, Dipartimento di Farmacia, Università degli Studi di Napoli Federico II, Via D. Montesano 49, 80131 Napoli, Italy; E-Mails: cimperat@unina.it (C.I.); filomena.daniello@unina.it (F.D.); aiello@unina.it (A.A.)

**Keywords:** meroterpenes, terpene quinone, terpene hydroquinone, sponges, ascidians, soft corals

## Abstract

Meroterpenes are widely distributed among marine organisms; they are particularly abundant within brown algae, but other important sources include microorganisms and invertebrates. In the present review the structures and bioactivities of meroterpenes from marine invertebrates, mainly sponges and tunicates, are summarized. More than 300 molecules, often complex and with unique skeletons originating from intra- and inter-molecular cyclizations, and/or rearrangements, are illustrated. The reported syntheses are mentioned. The issue of a potential microbial link to their biosynthesis is also shortly outlined.

## 1. Introduction

Quinones are ubiquitous in nature, occurring as secondary metabolites in many organisms; often they are molecules essential to life, being intimately related to the oxidative processes in cells [1]. Polyprenylated 1,4-benzoquinones and hydroquinones, such as ubiquinones, plastoquinones, and tocopherols, are widespread in plants and animals, in which they play important roles in electron transport, photosynthesis, and as antioxidants [1,2]. Terpene quinone/hydroquinone natural products differing from the above-mentioned groups frequently occur as secondary metabolites in many organisms; they form a class of complex metabolites, generally called meroterpenes, of mixed biosynthetic origin which are partially derived from terpenoids. In addition to their wide occurrence, meroterpenes display a huge range of structural diversity, with structures varying from simple compounds comprising a prenyl unit linked to the hydroquinone unit to unique architectural scaffolds, often linked with varied functionalities, arising from intra- and intermolecular ring closures and/or rearrangements of the terpene chains. Moreover, they display important biological activities, undoubtedly related to their most prominent chemical feature, that is their ability to undergo redox cycling to generate reactive oxygen species (ROS) which can damage cells [3,4].

In the marine environment, meroterpenes have been isolated mainly from brown algae and microorganisms, but another important source are marine invertebrates, mainly sponges and tunicates. The present review provides an update on the meroterpenes so far isolated from marine invertebrates; it describes the structures and biological activities of 300 natural products, thus highlighting the structural diversity generated in this class of natural products and their potential in drug discovery. The issue of a potential microbial link to their biosynthesis is also shortly outlined.

## 2. Meroterpenes from Ascidians

**Figure 1:**
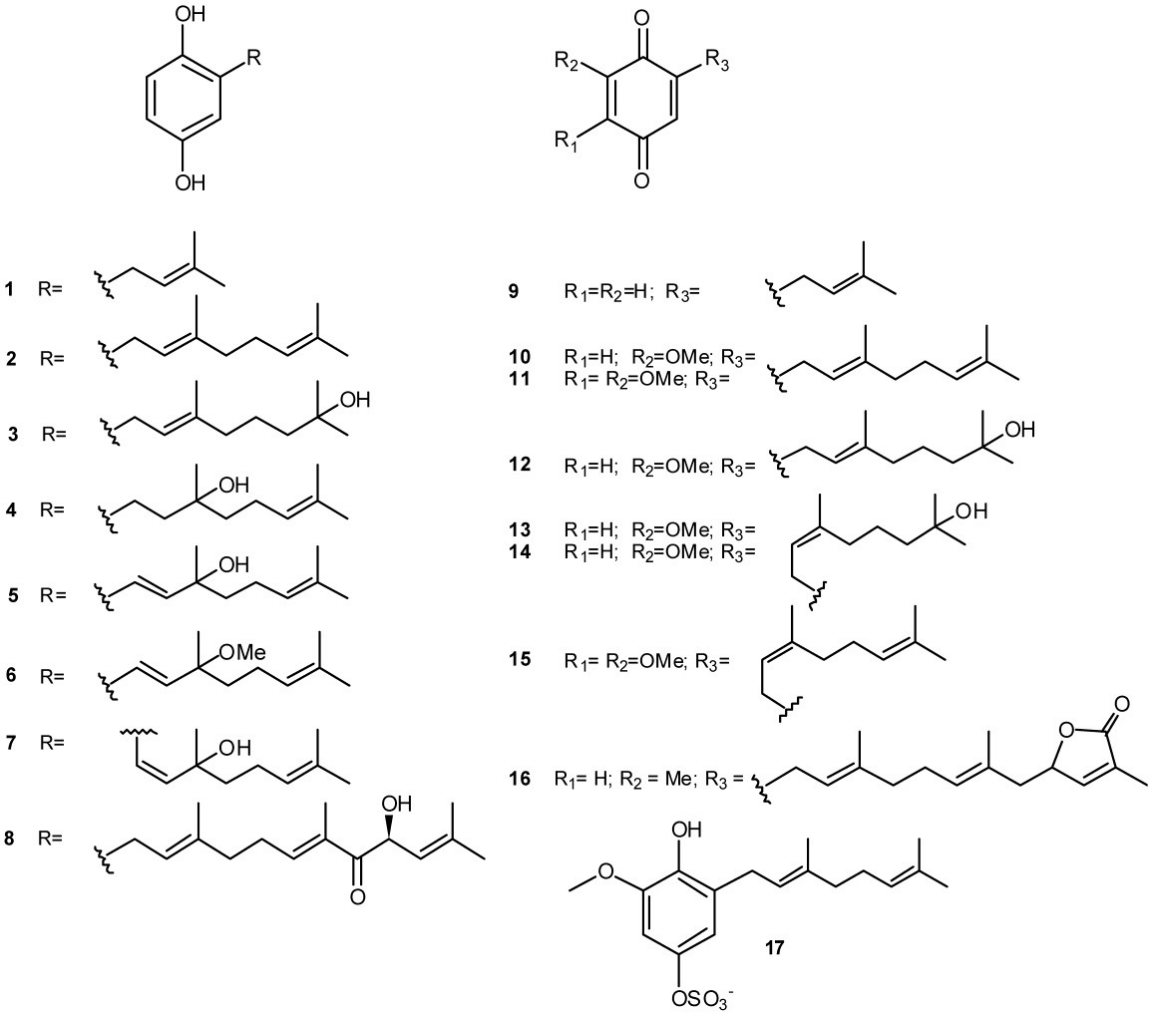
Quinones and hydroquinones linked with linear prenyl chains.

While the majority of metabolites isolated from ascidians are nitrogen-containing compounds (alkaloids or peptide related compounds), ascidians belonging to the genus *Aplidium* are known as a rich source of meroterpenes [5,6,7]. The first biologically active tunicate metabolite was indeed geranylhydroquinone (**2**), isolated from an *Aplidium* sp. and, successively, found in many others *Aplidium* species; it was shown to offer protection against leukemia and tumor development in test animals [8]. Several linear diprenylquinones/hydroquinones (compounds **1**–**17**, Figure 1) have been then reported from diverse *Aplidium* species (*A. multiplicatum* [9], *A. savigny* [10], *A. conicum* [11], *A. glabrum* [12], *A. scabellum* [13], *Aplidium* spp. [14,15]); the majority of them possess a linear side chain of the geranyl type with rare examples of neryl derivative such as verapliquinone D (**13**), verapliquinone B (**14**), and glabruquinone B (**15**). *A*. *californicum* has been the source of the simple monoprenyl derivatives **1** and **9** which were identified as anticancer and antimutagenic agents [16]. Glabruquinone A (3-demethylubiquinone Q2, **11**) is closely related to the ubiquinones, although lacking the methyl group in the quinoid moiety; it is not a cytotoxin but demonstrated good cancer preventive activity on JB6 Cl 41 cell transformation activated by epidermal growth factor (EGF). Structure-activity relationships studies on its synthetic analogs demonstrated that this activity depend on the length of the side chain and on the position of the methoxyl groups in the quinone part of the molecule [17]. *In vivo*, anticancer properties of **11** and its synthetic analogs, as well as the molecular mechanism of its action against tumor cells, have been examined; it was shown to inhibit the growth of the solid Ehrlich carcinoma in mice and to induce apoptosis in various human tumor cell lines [18]. Both 2-geranyl-6-methoxy-1,4-hydroquinone-4-sulfate (**17**) and the triprenylated (farnesyl) hydroquinone rossinone A (**8**) were found to be active in an anti-inflammatory assay, *in vitro*, with activated human peripheral blood neutrophils; they inhibit the superoxide production when either *N*-formyl-methionyl-leucyl-phenilalanine (fMLP) or phorbol myristate acetate (PMA) were used to activate the respiratory burst [13,15]. The monoprenylhydroquinone **1** and geranylhydroquinone **2** were also active in the same assay [15], indicating that prenyl quinones could indeed hold promise for the development of new anti-inflammatory agents.

Several interesting classes of cyclic, polycyclic, macrocyclic, and/or dimeric prenyl quinones/hydroquinones are reported to occur in marine ascidians. The biosynthetic origin of these compounds has been largely speculated about; they clearly arise from corresponding derivatives with linear prenyl chains that are usually isolated concomitantly from the natural source. *Ortho*-prenylated quinones can undergo different chemical transformations, ranging from cascade cyclization reactions involving carbocation species [19] to pericyclic reactions, such as electrocyclizations or cycloadditions, via *ortho*-quinones methide intermediates [20]. However, with the data available, it is not possible to argue whether the above mentioned transformations occur in the organism, prior to its extraction, either enzymatically (in nature dehydrogenase enzymes appear to catalyze such processes [21]) or not, or that they take place during isolation and/or chromatographic purification.

**Figure 2:**
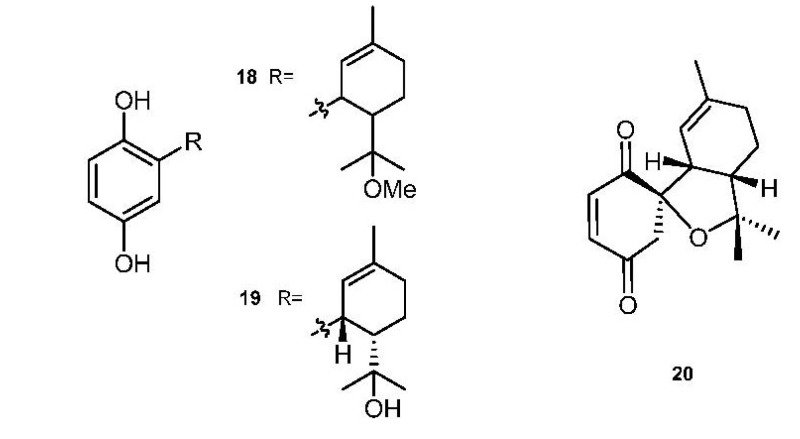
Cyclodiprenyl hydroquinones/quinones.

Cyclodiprenyl hydroquinones/quinones (Figure 2) have been isolated from *A. aff. densum* (methoxyconidiol, **18**) [22] and from *A. conicum* (conitriol, **19**, and conidione, **20**) [11]. Methoxyconidiol (**18**) displayed an antimitotic action on the first division of sea urchin embryos, disrupting M-phase progression and completely blocking cytokinesis without having any effect on DNA replication [23].

**Figure 3:**
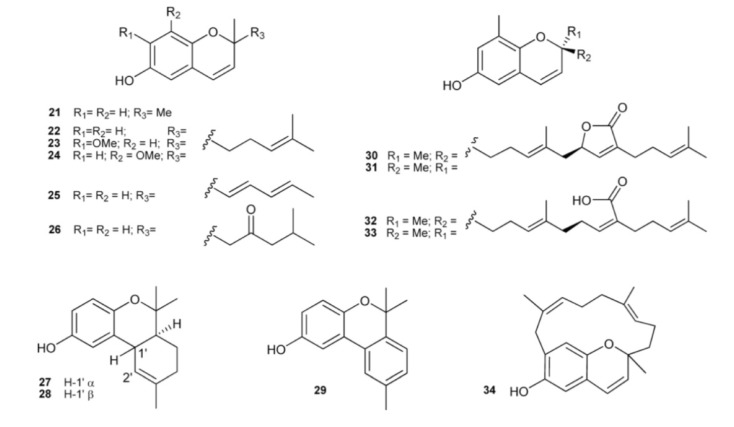
Chromene (2*H*-benzopyran) derivatives.

The occurrence of chromene (2*H*-benzopyran) derivatives as natural products has been reported in *A*. *californicum* (**21**) [14], *A. costellatum* (**22**) [24], *A. multiplicatum* (**23**) [9], *A. scabellum* (**24**) [13], and *A. solidum* (**25** and **26**) [25]. The chromane derivative **27** has been isolated from *Synoicum costellatum* [26], a species closely allied to the genus *Aplidium*, and successively found in *A. conicum* together with its C-1′ epimer, conicol (**28**) [11]. Didehydroconicol (**29**) has been isolated from *A. aff. densum* [22]. Tuberatolides (**30** and **31**) and sargachromenols (**32** and **33**), isolated from *Botryllus tuberatus* along with their putative linear precursor yezoquinolide (**16**), antagonized the chenodeoxycholic acid (CDCA)-activated human farnesoid X receptor (hFXR), a ligand-dependent transcription factor in the nuclear receptor superfamily which has been recently identified as a promising drug target in the treatment of atherosclerosis [27]. Longithorol E (**34**), from *A. longithorax*, is a unique macrocyclic chromenol containing a new 14 membered carbocycle [28] (Figure 3).

**Figure 4:**
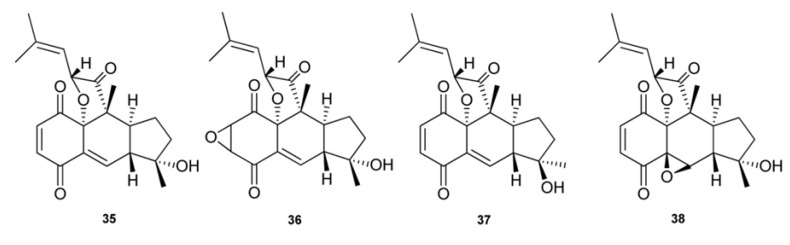
Cyclic triprenylated quinone/hydroquinones.

Rossinone B (**35**) is a triprenylated (farnesyl) quinone first isolated from an Antarctic *Aplidium* sp. [15] and successively recovered in the viscera extract of *A. fugiense*, also collected in Antarctica, along with the related compounds **36**–**38** [29] (Figure 4). Rossinone B has a rather novel structural architecture featured by a linearly fused 6-6-5-ring core, which so far has been found in only three plant-derived natural products, pycnanthuquinones A–C [19,30]. The tricyclic framework of **35** supposedly derive from the corresponding linear hydroquinone derivative rossinone A (**8**) which has been reported to co-occurr in *Aplidium* sp. [15]. Interestingly, neither acyclic hydroquinones nor putative quinone-containing precursors of compounds **35**–**38** were detected in *A. fugiense* extract [29]. Rossinone B (**35**) exhibited anti-inflammatory, antiviral, and antimicrobial activities [15]. Attracted by its novel chemical structure, promising biological properties and potentially intriguing biosynthetic pathway, a biomimetic total synthesis of (±)-rossinone B has been achieved through a highly efficient strategy featuring a series of rationally designed reactions, including an intramolecular vinyl quinone Diels-Alder reaction to construct the linear 6-6-5 tricyclic core of **35** [31].

**Figure 5:**
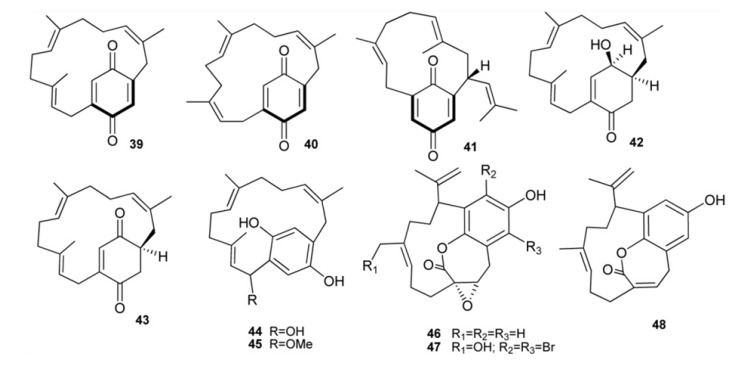
Monomeric meta-and paracyclophane type meroterpenes.

*A. longithorax* has been the source of longithorones and longithorols, a group of farnesylated quinone/hyroquinones featuring unprecedented macrocylic skeletons derived formally by the rarely encountered cyclization of farnesyl quinones/hydroquinones to give [9]- and [10]metacyclophane, as well as [12]paracyclophane structures [28,32,33,34,35]. Longithorones B–D (**39**–**41**), J (**42**), and K (**43**) together with longithorols C (**44**) and D (**45**), are monomeric C_21_ compounds (Figure 5). Longithorol C (**44**) could undergo an intramolecular cyclization, followed by dehydration, to yield the chromenol longithorol E (**34**), which is possibly an artifact of the isolation process. A short synthetic approach to the macrocyclic framework of longithorone C has been described via ring-closing metathesis using the Grubbs second generation catalyst [36]. Longithorone J (**42**) is the first example of a γ-hydroxy-cyclohexenone in this class of compounds. Floresolides A–C (**46**–**48**) are three further monomeric cyclofarnesylated hydroquinones isolated from an Indonesian *Aplidium* sp. They are unique members of the longithorone/longithorol class of meroterpenes, having an endocyclic ε-lactone bridging the aromatic ring and a [10]metacyclophane moiety. Floresolides showed moderate cytotoxicity against KB cells [37].

**Figure 6:**
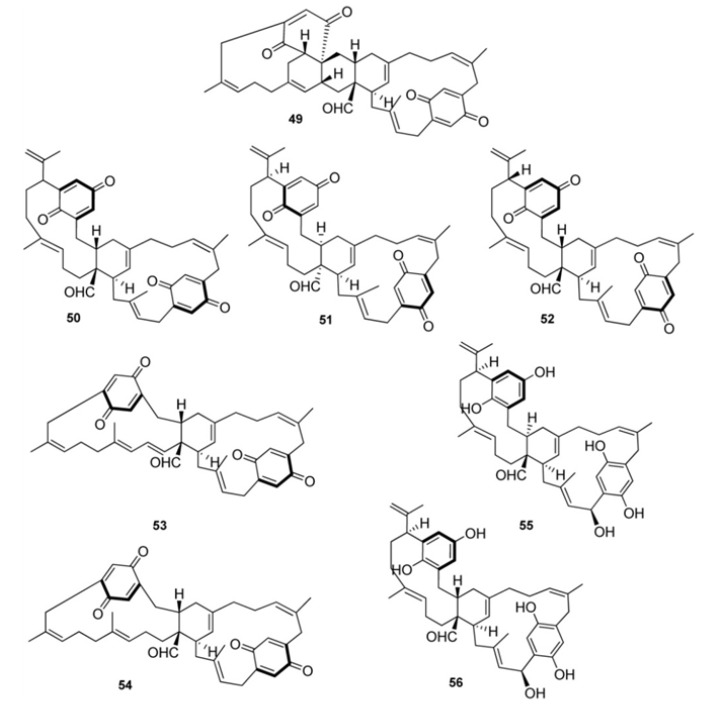
Dimeric paracyclophane type meroterpenes.

Longithorones A (**49**) and E–I (**50**–**54**) are dimeric compounds (Figure 6). Compounds **49**–**54**, as well as the monomeric longithorones **39**–**43**, exhibit atropisomerism arising from hindered rotation of quinone ring through their macrocyclic rings. The biosynthesis of dimeric longithorones, which have been supposed to originate by both intra- and intermolecular Diels-Alder reactions, has been speculated about. Fusion of the two farnesyl-quinone units can be envisioned as arising via a Diels-Alder cycloaddition of suitably unsaturated precursors, whereas rings B and C could arise by a transannular Diels-Alder reaction. The co-isolation of the monomers **39**–**43** provides some support for this proposal. The stereochemistry of the central carbocyclic rings in **35** and **39**–**43** is consistent with such a fusion [34]. An enantioselective biomimetic synthesis of longithorone A has been accomplished, which demonstrates the feasibility of the reactions proposed for the biosynthesis-albeit using non-enzymatic conditions. The challenge of a synthesis of longithorone A was heightened by the presence of two forms of chirality: the stereogenic centers present in the tricyclic core portion of the molecule and atropisomerism. The synthesis presents a unique example of chirality transfer in complex molecule synthesis involving the use of stereogenic centers to control atropisomerism, removal of the stereogenic centers, and transfer of the atropisomerism back to stereogenic centers in the natural product [38]. Longithorols A (**55**) and B (**56**) represent the first examples of hydroquinones in the [12]paracyclophane structure class. They were isolated as their pentaacetates because of their rapid decomposition occurring under purification conditions [35]. To date, the only biological activity reported for longithorones/longithorols class of marine metabolites pertains to longithorone A (**49**), which was shown to display cytotoxicity against P388 murine leukaemia cells.

**Figure 7:**
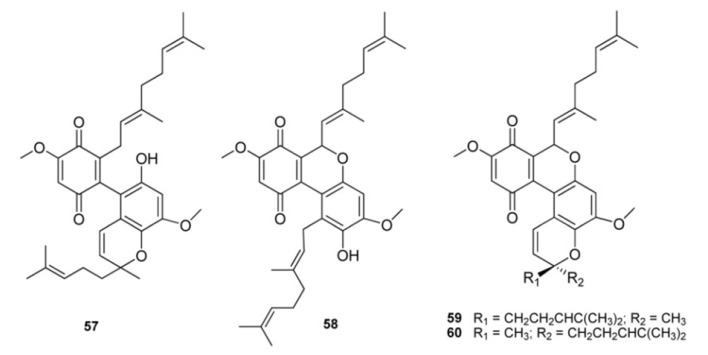
Dimeric benzo[*c*]chromene-7,10-dione containing meroterpenes.

Further examples of pseudodimeric meroterpenoids are scabellones A–D, (**57**–**60**) isolated from *A. scabellum* [13], possessing a benzo[*c*]chromene-7,10-dione scaffold particularly rare among natural products (Figure 7). Scabellone B (**58**) was found to inhibit the superoxide production by PMA-stimulated human neutrophils *in vitro*; it was also evaluated against the neglected disease parasites targets *Trypanosoma brucei rhodesiense*, *T. cruzi*, *Leishmania donovani*, and *Plasmodium falciparum* and exhibited selectivity toward *Plasmodium falciparum* (K1 chloroquine-resistant strain) with IC_50_ 4.8 μM and only poor cytotoxicity (L6 rat myoblast cell line, IC_50_ 65 μM) [13].

**Figure 8:**
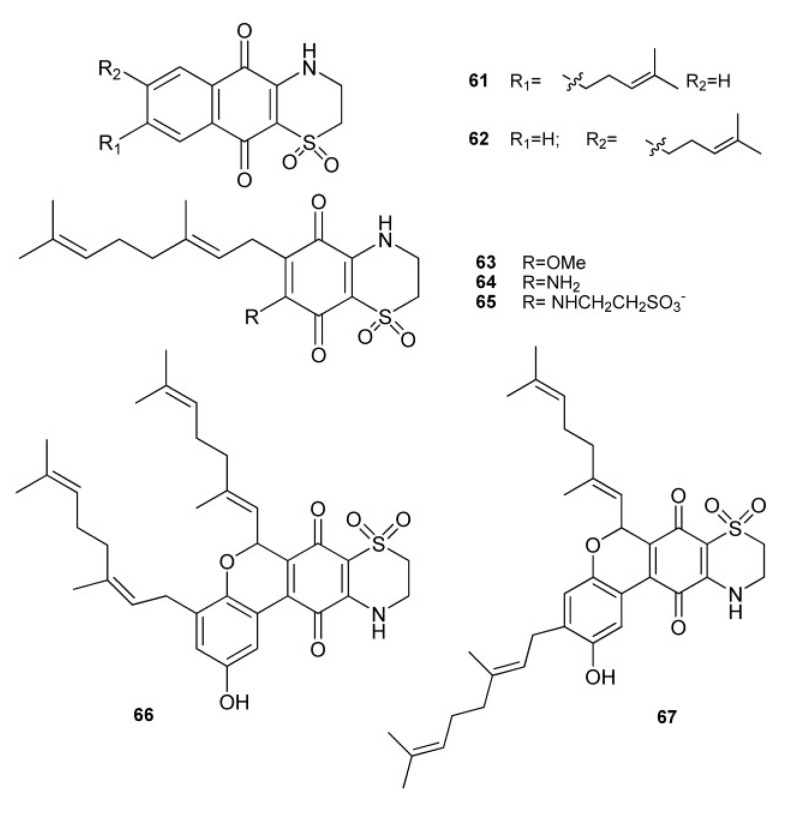
Prenylated thiazinoquinones from *A. conicum*.

A sample of *A. conicum* collected along Sardinia coasts gave rise to the isolation of a large group of unique prenylated thiazinoquinones, namely conicaquinones A and B (**61** and **62**) [39], aplidinones A–C (**63**–**65**) [40], and thiaplidiaquinones A and B (**66** and **67**) [41] (Figure 8). All these meroterpenes feature an unusual 1,1-dioxo-1,4-thiazine ring fused with the quinone portion. Aplidinones A–C (**63**–**65**) and conicaquinones A and B (**61** and **62**) possess a benzoquinones and a naphtoquinone moiety, respectively. Thiaplidiaquinones A and B (**66** and **67**) possess an unprecedented tetracyclic core, visibly composed of two geranylated benzoquinones that have fused together, whose biosynthetic origin has been speculated to stem from hypotaurine addition to tricyclic pyranoquinones derived from oxa-6π-electrocyclization of *ortho*-quinone methide tautomers of bis-benzoquinones [42]. Based on this premise, two biomimetic synthesis of the thiaplidiaquinones scaffold have been reported [42,43]. In a first concise total synthesis of thiaplidiaquinone A, the key ring forming steps are both based on biosynthetic considerations and involve the construction of the central benzo[c]chromene quinone unit by an extremely facile oxa-6π-electrocyclic ring closure reaction of an *ortho*-quinone intermediate, derived by tautomerization of a bis-benzoquinone, readily accessed from two simple phenolic precursors. This is followed by the installation of the 1,4-thiazine-dioxide ring by reaction of the benzo[c]chromene quinone with hypotaurine [43]. An alternative biomimetic synthesis of both thiaplidiaquinones A and B has been reported where bis-benzoquinones construction, instead of via a Suzuki-Miyaura reaction [43], was achieved simply by base-mediated dimerization of geranylbenzoquinone. Subsequent reaction with hypotaurine yielded the dioxothiazine regioisomers of thiaplidiaquinones A and B [42]. Both conicaquinones A and B showed a marked and selective cytotoxic effects on rat glioma cells [40], and thiaplidiaquinones were strongly cytotoxic against Jurkat cell line, derived from a human T lymphoma, inducing cell death by apoptosis [41]. The pro-apoptotic mechanism of thiaplidiaquinones involves the induction of a strong production of intracellular reactive oxygen species (ROS) in the cells, likely due to the inhibition of the plasma membrane NADH-oxidoreductase (PMOR) system, an important target for anticancer drugs, through interference with the coenzyme-Q binding site [41]. In order to validate the structural assignment made for aplidinones by theoretical means a synthetic approach has been undertaken which yielded some synthetic analogs of aplidinone A in which the geranyl chain is replaced by other alkyl chains [44]; these compounds as well as the natural metabolite **63** were subjected to cytotoxicity assays and preliminary structure-activity relationships (SAR) studies. Both aplidinone A and its synthetic analogs were shown to possess interesting cytotoxic effects; SAR studies revealed that cytotoxic activity depends on the nature and the length of side chain linked to the benzoquinone ring and, mainly, on its position respect to the dioxothiazine ring. The study also evidenced one of the synthetic analogs as a potent cytotoxic and pro-apoptotic agent against several tumor cell lines, which also inhibits the TNFα-induced NF-κB activation in a human leukemia T cell line [44]. 

## 3. Meroterpenes from Sponges

Marine sponges have yielded a huge variety of meroterpenes having a terpenoid skeleton varying from sesqui-, di-, sester- or triterpene units.

The large class of sesquiterpene quinones isolated from various species of marine sponges, has attracted the attention of researchers because of their biological properties, including antimicrobial [45], antileukemic [46], anti-malarial [47], immunomodulatory [48,49], and anti-HIV [50] activities. Above all, the cytotoxic and antiproliferative properties of many sesquiterpene quinones/hydroquinones isolated from sponges have supported several studies for the development of new antitumor agents [51,52].

**Figure 9:**
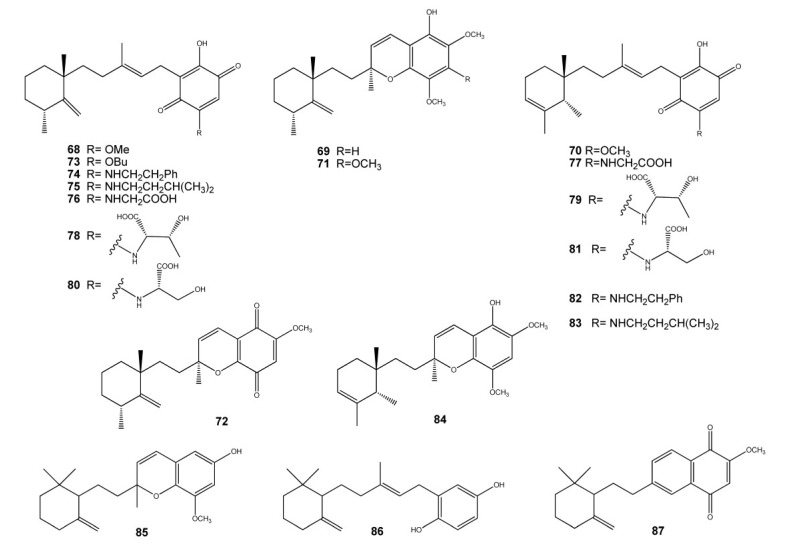
Metachromins.

A large family of antineoplastic compounds, named metachromins (**68**–**87**), has been isolated from sponges of the genus *Spongia*, *Thorecta* and *Hippospongia* (Figure 9) [53,54,55,56,57,58]. They exhibited potent antitumor activity against L1210 murine leukemia cells *in vitro*. Metachromins A–C (**68**–**70**) showed also potent coronary vasodilating activity, markedly inhibiting KCl (40 mM) induced contraction of the rabbit isolated coronary artery with an IC_50_ value of 3 × 10^−6^ M each. While metachromins D–H (**71**–**75**) exhibited cytotoxity against human epidermoid carcinoma KB cells with IC_50_ values of 10, 0.4, 1.9, >10, and 6.4 μg/mL, respectively. Metachromins L (**76**), M (**77**), S (**83**), and T (**84**) showed cytotoxicity against L1210 murine leukemia (IC_50_, 4.0, 3.5, 5.2 and 3.0 μg/mL, respectively) and KB human epidermoid carcinoma cells (IC_50_, 4.0, 5.4, >10 and 5.6 μg/mL, respectively) *in vitro*, while metachromins N–R (**78**–**82**) did not show such activity (IC_50_ > 10 μg/mL). Metachromins U–W (**85**–**87**) were screened against four human tumor cell lines [MCF–7 (breast), SF-268 (CNS), H-460 (lung), and HT-29 (colon)], as well as a mammalian cell line [Chinese hamster ovary (CHO-K1) cells]. All three compounds were found to be cytotoxic against all cell lines, with **86** being the most active. Surprisingly, **87**, possessing a napthoquinone functionality, which is known to impart significant cytotoxic properties to various molecules [58], was significantly less active than both **85** and **86**. Interestingly, metachromins N–R (**78**–**82**) [57], which contain both quinone and phenol functions, are inactive, most likely due to the bulky nature of the substituents present on the quinone portion.

In fact, most of sesquiterpene benzo(hydro)quinone isolated from sponges possess a decalin structure, consisting of a drimane or 4,9-friedodrimane skeleton, connected to the quinone/hydroquinone moiety generally via one carbon-carbon bonding. Particularly prolific sources of this sort of meroterpenes are marine sponges belonging to the *Dysidea* genus.

**Figure 10:**
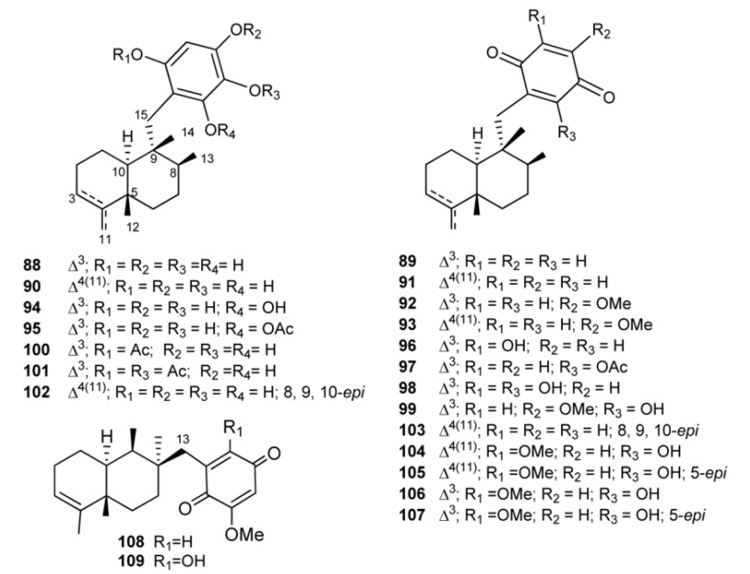
4,9-Friedodrimane-type skeleton containing meroterpenes of the avarol/avarone series.

Compounds having a typical 4,9-friedodrimane skeleton are shown in Figure 10. Avarol (**88**) and its quinone derivative avarone (**89**) have been isolated from the marine sponge *Dysidea avara* [59,60]; the absolute stereochemistry of **88**, which has been stated by spectroscopic and chemical methods in 1976 [61] was later confirmed by crystallographic analysis [62]. Both compounds were discovered as anti-leukemia agents *in vitro* and *in vivo* [46,63,64,65,66], and later it was found that they had an inhibitory capacity *in vitro* against HIV-1 [66,67,68]. Various formulations with high avarol content have been used for the prevention and treatment of psoriasis, dermatitis, skin cancer, tumors in the gastrointestinal tract, urinary tract, and viral infection [69]. Their potent T-lymphotropic cytostatic activity, low toxicity in mice, the ability to cross the blood-brain barrier and the capacity to stimulate the synthesis of interferon make both these compounds optimum candidates for transformations aimed at improving their cytostatic and antiviral activity [63,64,65,66,67,68,69].

A number of derivatives of avarol and avarone have been isolated from other *Dysidea* species. Examples are neoavarol (**90**), neoavarone (**91**), 4′-methoxyavarone (**92**) and 4′-methoxyneoavarone (**93**), isolated from a sample of *Dysidea* sp. collected in Okinawa [70], and 6′-hydroxyavarol (**94**), 6′-acetoxyavarol (**95**), 3′-hydroxyavarone (**96**), 6′-acetoxyavarone (**97**), 3′,6′-dihydroxyavarone (**98**) and 6′-hydroxy-4′-methoxyavarone (**99**), isolated from *Dysidea cinera* [71]. Some of these compounds showed cytotoxic activity, 3′-hydroxyavarone (**96**), 6′-acetoxyavarol (**95**) and 3,6′-dihydroxyavarone (**98**) exhibiting high potency against P-388 mouse lymphoma. From different extracts of *Dysidea avara* collected from different places (Japan, the Solomon Islands, and others), mono-(**100**), diacetyl-(**101**), and 6′-hydroxy-5′-acetylavarol have been isolated [49,72,73]. A number of avarol derivatives exhibited interesting activity in enzyme assays measuring inhibition of various functions of HIV-1 reverse transcriptase [74].

From the Pacific sponge *Dysidea arenaria*, a pair of mildly cytotoxic compounds, arenarol (**102**) and arenarone (**103**), have been isolated having the same rearranged sesquiterpene skeleton as avarol, but with *cis*- rather than *trans*-decalin stereochemistry [75].

Ilimaquinone (**104**), first isolated from the Hawaiian sponge *Hippospongia metachromia* [76] and successively recovered in several other sponge species along with its 5-*epi*-analogous (**105**) and both isospongiaquinone (**106**) and 5-*epi*-isospongiaquinone (**107**) [77,78,79,80]. In 1987 Capon reversed the absolute stereochemistry of (−)ilimaquinone, placing (+)arenarol and (−)ilimaquinone in the same 8*S*, 9*R* absolute stereochemical series [81]. Several important activities have been reported for ilimaquinone, including anti-HIV activity [50] and ability to protect cells from the toxic effects of ricin and diphtheria toxin [82]. Particularly, it has been suggested that **104** induces a concentration-dependent antiproliferative effect in several types of cancer cell lines. The anticancer mechanism of ilimaquinone in the representative PC-3 cells was identified; it induces a time-dependent increase in G1 phase arrest and a subsequent increase in the hypodiploid sub-G1 phase (apoptosis) of the cell cycle. Although ilimaquinone-induced Golgi vesiculation [83,84], the data showed that the inhibition of cancer cell growth did not occur through Golgi fragmentation. Ilimaquinone (**104**) also inhibited the DNA binding of NF-κB; however, this inhibitory effect cannot explain the ilimaquinone-induced anticancer effect. In brief, it is suggested that ilimaquinone (**104**) induces its antiproliferative effect through the G1 arrest of the cell cycle and the up-regulation and nuclear translocation of CHOP/GADD153 [85]. 5-*epi*-Ilimaquinone (**105**) showed cytotoxic activity against P-388 leukemia cells (2.2 μg/mL) and different solid tumors: A-549 (0.9 μg/mL), HT-29 (3.4 μg/mL) and B16/F10 (1.1 μg/mL) [80].

Bolinaquinone (**108**) is a cytotoxic sesquiterpene isolated from a *Dysidea* sponge in which quinone moiety is located on an unusual carbon of the 4,9-friedodrimane skeleton [86]. This compound was cytotoxic against HCT-116 human colon carcinoma (IC_50_ = 1.9 μg/mL) and it has been demonstrated to act by interfering with or damaging DNA. 21-dehydroxybolinaquinone (**109**), isolated from the Hainan sponge *Dysidea villosa*, showed moderate PTP1B inhibitory activity and cytotoxicity, with IC50 values of 39.5 and 19.5 mM, respectively [87].

**Figure 11:**
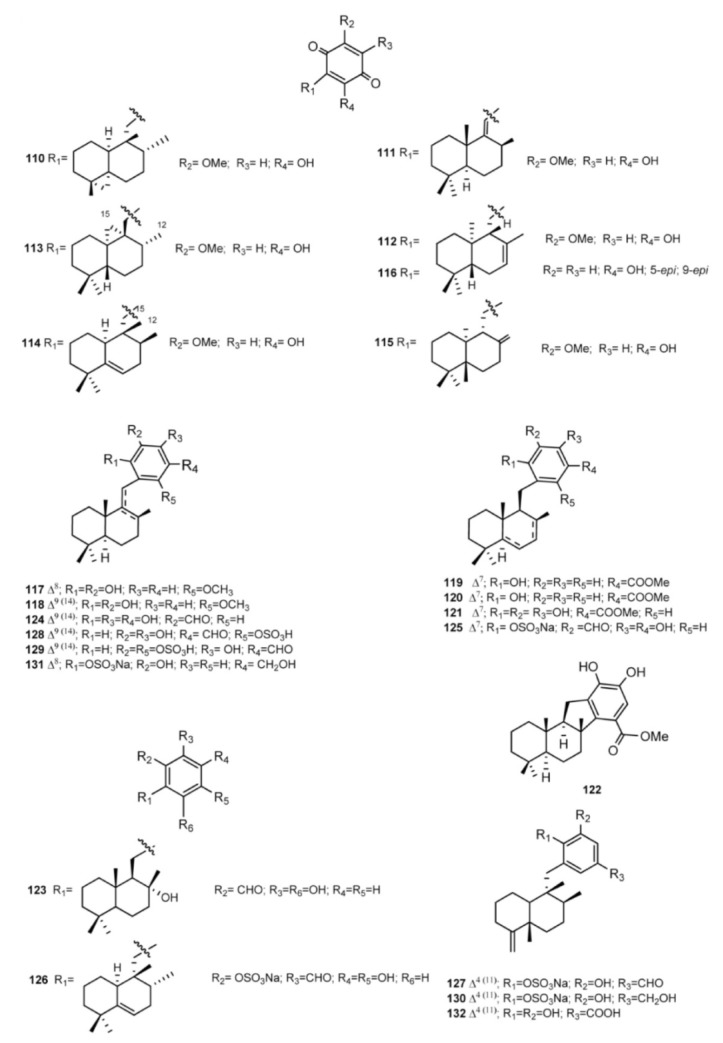
Drimane-type skeleton containing meroterpenes.

Examples of meroterpenes of the drimane series (Figure 11) are dactylospongiaquinone (**110**) and spongiaquinone (**111**), isolated from the sponge *Dactylospongia* n. sp. [79,88], isohyatellaquinone (**112**), dictyoceratidaquinone (**113**), mamanuthaquinone (**114**), and hyatellaquinone (**115**) from both *Dactylospongia* and *Fasciospongia* sponges [89,90,91,92]. Total synthesis of both hyathellaquinone (**115**) and spongiaquinone (**111**) has been reported [93,94]; particularly, synthesis of (−)-hyatellaquinone led to revision of absolute configuration of the naturally occurring (+)-isomer [93]. The anti-inflammatory sesquiterpene-quinone **116** has been isolated from the New Zealand sponge *Dysidea* cf. *cristagalli* [95].

Wiedendiols A (**117**) and B (**118**) were isolated from the marine sponge *Xestospongia wiedenmayeri*, collected in the Bahamas [96]. The CETP-SPA inhibition assays carried out with these compounds revealed an IC_50_ = 5 μM in both cases. Later, the inhibition of CETP was verified using a precipitation method to separate lipoproteins after incubation of HDL radiolabeled with LDL and CETP. In this assay, both **117** and **118** had an IC_50_ of 1.0 and 0.6 μM, respectively. Wiedendiol B is a ten-fold stronger inhibitor of cyclooxigenase-2 than the reference compound indomethacine [97].

Smenodiol (**119**), isolated from a Seychellean sponge belonging to the genus *Smenospongia* [98], and compounds **120** and **121**, isolated from *Dactylospongia elegans* [80], feature a carboxilate function on the aromatic ring; smenodiol could be considered a possible direct precursor of pelorol (**122**), isolated from *D. elegans* [99], as it is a simple cyclization product thereof (Figure 11).

An aldehyde function is the distinctive feature of siphonodictyals A–D (**123**–**126**), G (**127**), B2 (**128**), and B3 (**129**), which have been isolated from Siphonodictyonspecies along with the relevant alcohols siphonodictyols G (**130**) and H (**131**) and siphonodictyoic acid (**132**); most of them were sulfated [100,101,102]. The isolated compounds were tested for antimicrobial activity (antibacterial, antifungal); it has been suggested that the different substituents on the aromatic moieties have an impact on activity and that the *ortho*-hydroquinone structure may be the active center of the molecules. It is likely that the hydroquinone is oxidized in the metabolism of the assay organisms to yield the more toxic *ortho*-quinone.

**Figure 12:**
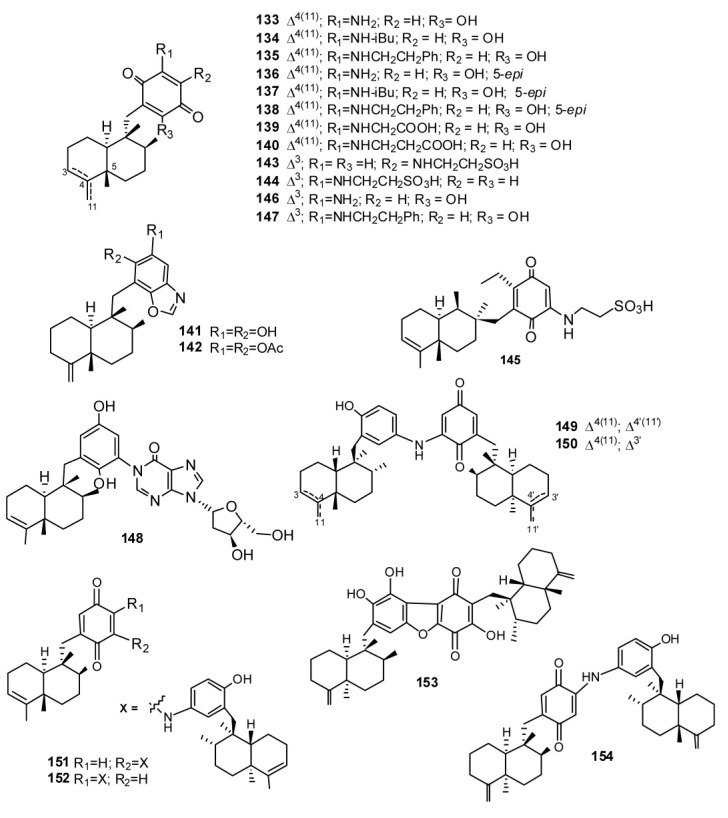
Amino- and amino acid- substituted quinone containing meroterpenes.

**Figure 13:**
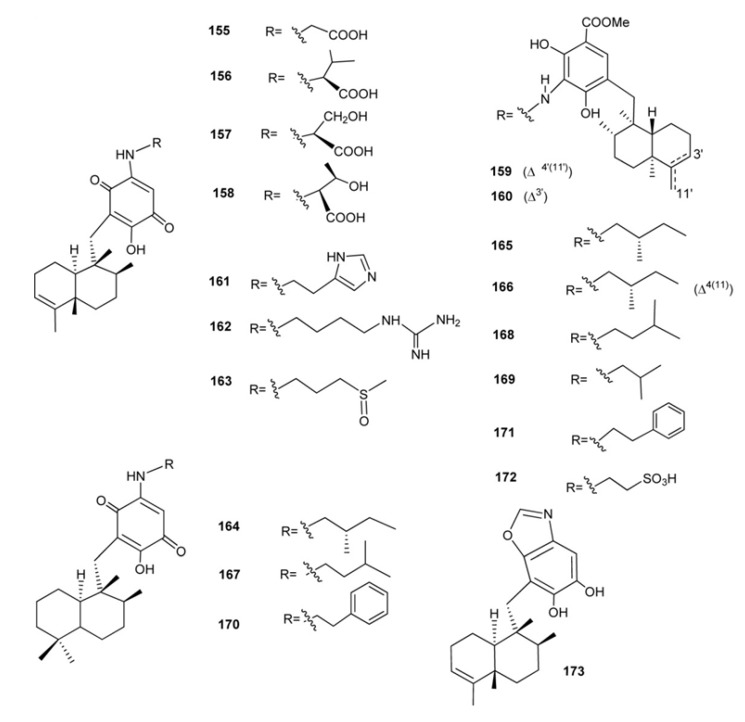
Nakijiquinones and nakijinol.

The presence of aminoquinone compounds is not very common in natural products; however, several sesquiterpenes quinones/hydroquinones, with such a drimane or a 4,9-friedodrimane, where the aromatic fragment is substituted with simple amines and amino acids have been isolated from sponges (Figure 12, Figure 13). The sesquiterpene aminoquinones smenospongine (133), smenospongiarine (134), smenospongidine (135), and their corresponding 5-epimers 136–138, have been isolated from different sponge species [47,70,103,104]. Erythroid differentiation of K562 cells induced by compounds **133**–**138** and other related compounds has been studied. On the basis of structure–activity relationship studies, the following evidences were obtained: (a) the quinone structure is indispensable; (b) the amino group should play an important role; (c) the substituents at the amino group are not crucial; (d) the configuration at the C-5 in sesquiterpene part is not important [105,106]. Smenospongines B (**139**) and C (**140**) were isolated from *D.* *elegans* collected in Australia along with the sesquiterpene benzoxazole nakijinol B (**141**) and its diacetyl derivative **142** [107]. The biological activities of these compounds were established against a panel of human tumor cell lines, as well as a normal mammalian cell line. The compounds were found to have cytotoxic activities in the range 1.8–46 μM and appeared to lack selectivity for tumor versus normal cell lines. The presence of two bulky acetate moieties resulted in an approximate two-fold increase in the activity of **142** compared to the diol **141**. One possible explanation for this increase in activity is that the acetate groups may contribute to greater bioavailability through enhanced membrane permeation after which metabolism, possibly hydrolysis by esterases, releases the active compounds intracellularly [108]. For **139** and **140**, the additional methylene in the nitrogen-substituted side chain had the effect of reducing observed activity by a factor of 2 (Figure 12).

From different extracts of *Dysidea avara* collected from different places (Japan, the Solomon Islands, and others), melemeleone A (**143**), melemeleone B (**144**) have been isolated possessing a taurine moiety linked to the quinone ring. Melemeleone B (**144**) was proved to have a certain activity against PTK (Protein Tyrosine Kinase) pp60^v-sarc^ (dose: 20 μg/mL) with an IC_50_ = 28 μM [49]. Dysidine (**145**), found in the Hainan sponge *Dysidea villosa*, also features a taurine residue on the aromatic portion, which is otherwise connected at C-11 of the decalin ring [109]. Compound **145** effectively activated the insulin signaling pathway, greatly promoted glucose uptake in 3T3-L1 cells, and showed strong insulin-sensitizing activities. The potential targets of action for dysidine were probed, and the results indicated that dysidine exhibited its cellular effects through activation of the insulin pathway, possibly through the inhibition of protein tyrosine phosphatases, with more specific inhibition against protein tyrosine phosphatase 1B (PTP1B) [110]. 3′-Aminoavarone (**146**), and 3′-phenethylaminoavarone (**147**), have been isolated from the marine sponge *Dysidea* sp. collected in Papua New Guinea along with avinosol (**148**), which is the first example of a naturally occurring meroterpenoid-nucleoside conjugate. Avinosol showed antiinvasion activity in a cell-based assay [111]. The structures of popolohuanones A–F (**148**–**154**), isolated from different species of the genus *Dysidea* [66,112,113,114], are formed by two subunits. Popolohuanone A (**149**) and popolohuanone F (**154**) showed DPPH radical scavenging activity, with an IC_50_ value of 35 μM [114]. Popolohuanone E (**153**) was revealed as a potent topoisomerase II inhibitor with selective cytotoxicity against the A549 non-small cell human lung cancer cell line [113] (Figure 12).

Nakijiquinones A–R (**155**–**172**) and nakijinol (**173**) (Figure 13) constitute a large class of sesquiterpenequinones of natural origin with an amino acid (nakijiquinones A–D, **155**–**158**) an heterocyclic moiety (nakijinol, **173**), or an amino acid-derived amino group (nakijiquinones G–R, **161**–**172**) which have been isolated from five collections of Okinawan marine sponge of the Family Spongiidae [115,116,117,118,119]. Nakijiquinones E (**159**) and F (**160**) were the first dimeric sesquiterpeneoids possessing a 3-aminobenzoate moiety [117]. Nakijiquinones A–D (**155**–**158**) showed *in vitro* cytotoxicity against L-1210 (IC_50_ values between 2.8 and 8.1 μg/mL) and KB (IC_50_ values between 1.2 and 7.6 μg/mL [115], while nakijiquinones G–I (**143**–**145**), slightly cytotoxic, and C (**141**) showed inhibitory activity against protein tyrosine kinase HER2 [116,118]. Studies on the total synthesis and structure-activity relationship of nakijiquinones have been performed by Waldmann *et al.* and it was found that simplified analogs of nakijiquinones A–D exhibited inhibitory activities against different kinds of tyrosine kinases [120]. Nakijiquinones J–R at 1 mM were tested for inhibitory activities against EGFR and HER2 tyrosine kinases. Among them, nakijiquinones P and R exhibited inhibitory activities against EGFR (76 and >99% inhibition, respectively), while nakijiquinones N, O and R showed inhibitory activities against HER2 (66%, 59% and 52% inhibition, respectively). The HER2/Neu tyrosine kinase receptor is hugely overexpressed in about 30% of primary breast, ovary, and gastric carcinomas. Nakijiquinones are the only naturally occurring inhibitors of this important oncogene [51,120].

**Figure 14:**
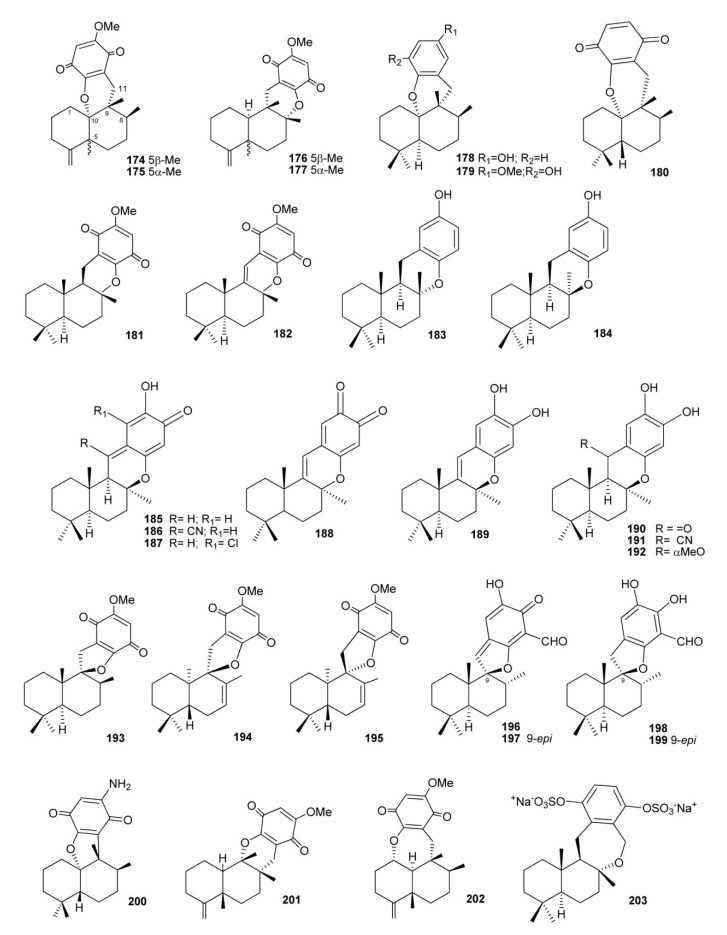
Meroterpenoids with a fourth ring originated by an ether linkage between the benzo(hydro)quinone and terpene moieties.

There are some compounds that also have a fourth ring, through an oxygen (most times) or carbon bridge between the decalin and the benzo(hydro)quinone ring (Figure 14). Dactyloquinones A and B(**174** and **175**), having a six-membered-ring made by ether linkages between C-10 and C-17 of ilimaquinone and 5-epi-ilimaquinone, respectively [121], dactyloquinones D and E (**176** and **177**), both possessing a six membered-ring made from ether linkages between C-8 and C-17 of of ilimaquinone and 5-epi-ilimaquinone, respectively [122], have been isolated from a collection of *Dactylospongia elegans* at Okinawa (Japan). An oxygen bridge between the benzo(hydro)quinone moiety linked at C-11 and C-8 or C-10 to form dihydropyran rings has been also observed in aureol (**178**) [123], strongylin (**179**) [124], smenoqualone (**180**) [45], cyclospongiaquinone-1 (**181**) [79,88], dehydrocyclospongiaquinone-1 (**182**) [79,88], and *ent*-chromazonarol (**183**), isolated from *Dysidea pallescens* [125], whose structure was confirmed by chemical synthesis performed from (−)-scalareol [126]. The epimer of **183**, 8-epichromazonarol (**184**), was isolated from *Smenospongia aurea* [123]. Interestingly, *ent*-chromazonarol (**183**) resulted moderately cytotoxic activity against P-388, A-549. HT-29 and MEL-28 cells, while its enantiomer chromazonarol, isolated from a brown alga, was inactive [127]. An enantioselective cyclization of 2-(polyprenyl)phenol derivatives to afford polycyclic terpenoids bearing a chroman skeleton such as (−)-chromazonarol by a new artificial cyclase has been described [128]. Puupehenone (**185**) possessing a drimane skeleton differs from typical natural sesquiterpene quinones by having a quinone–methide system. First isolated by Scheuer and co-workers from the sponge tentatively identified as *Chondrosia chucalla* [129], **185** has so far been isolated from different sponges, mainly of orders Verongida and Dictyoceratida, together with many other puupehenone-derived congeners (**186**–**192**) engendered by the presence of the highly electrophilic quinone–methide system and oxygen functionalities [129,130,131,132,133,134,135,136,137] (Figure 11). The puupehenones display a wide range of biological properties as angiogenesis inhibitors [138], antitumor [130,131], antifungal [48,129], antiviral [48], antimalarial [130], antituberculosis [139], immunomodulatory [138,140], and antioxidant agents [141]. The total synthesis of (+)-puupehenone (**185**) was achieved in ten steps starting from commercially available (+)-sclareolide [142].

Structures with a fourth five-membered oxygen spiranic ring are cyclospongiaquinone-2 (**193**) [79,88], its analogs 7,8-dehydrocyclospongiaquinone-2 (**194**) and 9-epi-7,8-dehydrocyclospongiaquinone-2 (**195**) [89], and the corallidyctals A–D (**196**–**199**), isolated from the marine sponge *Aka* (=*Siphonodictyon*) *coralliphagum* [102,143]. Both corallidictyal A (**196**) and B (**197**) inhibit PKC with an IC_50_ = 28 μM, while assays addressing another cAMP-dependent kinase did not afford inhibition at concentrations of 300 μM, indicating its selectivity. Further, the assays revealed selectivity against the α isoform of PKC [102]. Corallidictyals C (**198**) and D (**199**) were tested in antiproliferative assays using cultures of mouse fibroblasts and activity was linked to the presence of the *ortho*-hydroquinone moiety [143]. Uncommon cyclizations are observed in the aminoquinone cyclosmenospongine (**200**) [144,145], neodactyloquinone (**201**) [146], and dactyloquinone C (**202**) [122]; this latter compounds, together with bis(sulfato)-cyclosiphonodictyol A (**203**), isolated from Siphonodictyon coralliphagum, represent the only examples with a further seven-membered ring. Compound **203** showed inhibitory activity against the binding of [3H]-LTB_4_ to human neutrophils, with IC_50_ = 44.5 μM [147] (Figure 14).

**Figure 15:**
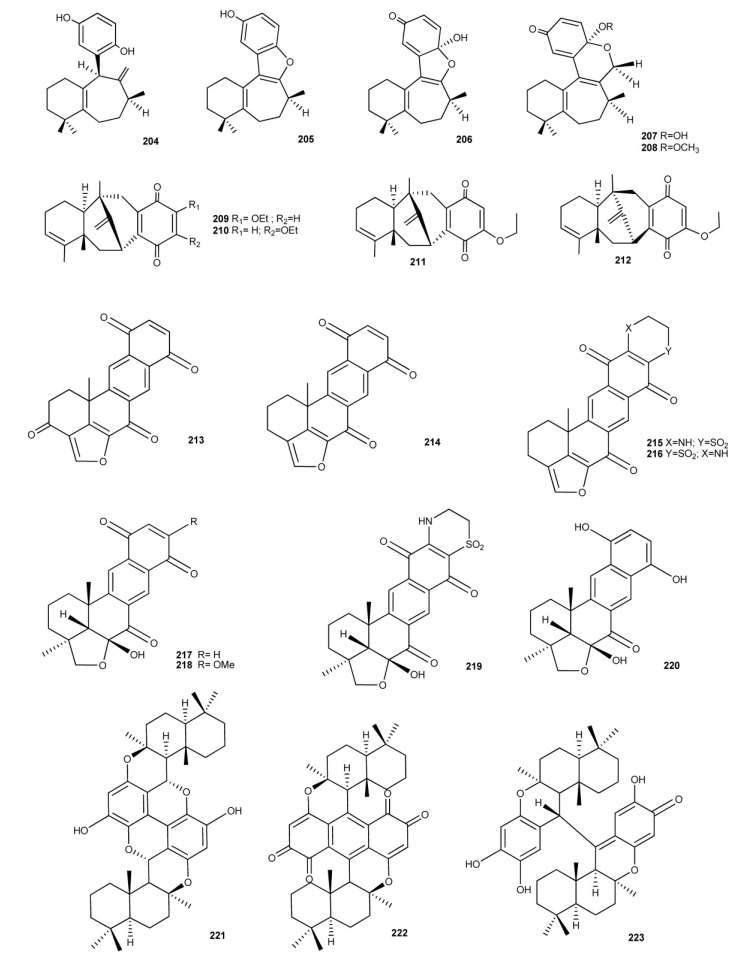
Sesquimeroterpenoids with unusual and/or complex structures.

Sesquiterpene benzo(hydro)quinones with more unusual and/or complex structures are showed in Figure 15. Frondosins A–E (**204**–**208**), from *Dysidea frondosa*, were found to be inhibitors of interleukin-8 receptors and protein kinase C in the low micromolar range [148].

Dysidavarones A–D (**209**–**212**), isolated from *Dysidea avara*, possess the unprecedented “dysidavarane” carbon skeleton. Their cytotoxic activity against four human cancer cell lines, cervix (HeLa), lung (A549), breast (MDA231), and hepatoma (QGY7703), were evaluated. In particular, Dysidavarones A (**209**) showed a growth inhibitory effect against HeLa cells with an IC_50_ value of 39.9 μM, and dysidavarone D (**212**) showed inhibitory effects against the four cell lines with IC_50_ values of 28.8, 21.4, 11.6, and 28.1 μM, respectively. In addition, dysidavarones A (**209**) and D (**212**) also showed inhibitory activity on protein tyrosine phosphatase PTP1B with IC_50_ values of 9.98 and 21.6 μM, respectively [149].

Investigation of Pacific sponges of the genus *Xestospongia*, yielded some quinone compounds with biological activites, halenaquinone (**213**) and xestoquinone (**214**) [150,151]. Both compounds showed cardiotonic properties and inhibitory activity against Ca^2+^ ATPase, phosphatidylinositol 3-kinase, protein-tyrosine kinase, and mammalian topoisomerase I. In addition, halenaquinone (**213**) showed cytotoxic activity against KB and P388 cell lines and it inhibited recombinant human Cdc25B *in vitro* with IC_50_ values of 0.7 μM [152,153]. From an *Adocia* sp. sponge from Truk Lagoon were isolated adociaquinones A (**215**) and B (**216**) [154]. They exihibited a cytotoxic activity toward P388, HCT, KB16, and HEP-3B cell lines. Moreover, compound **216** inhibited recombinant human Cdc25B *in vitro* with IC_50_ values of 0.07 μM [152].

Alisiaquinones A–C (**217**–**219**) and alisaquinol (**220**) [155], isolated from a New Caledonian deep water sponge, are related to xestoquinone, halenaquinone, adociaquinones, but they show an unusual substitution pattern on the furan ring. These new meroterpenes displayed mild activity with micromolar range on two enzymatic targets of importance for the control of malaria, the plasmodial kinase Pfnek-1 and a protein farnesyl transferase (PFT) as well as on different chloroquine-sensitive and chloroquine-resistant strains of *Plasmodium* *falciparum* [155].

Particularly, alisiaquinone C (**215**), bearing the taurine substituent, displayed a submicromolar activity on *P.* *falciparum* and a competitive selectivity index on the different plasmodial strains, especially on the chloroquine-resistant strain PfFcMC29. For alisiaquinones A (**217**) and C (**219**), the *in* *vivo* activity was also investigated, but they displayed a relatively high level of toxicity. Bispuupehenone (**221**), dipuupehedione (**222**), and diplopuupehenone (**223**) are dimeric molecules isolated from *Dysidea* sp. and *Hyrtios* sp. [131,137,141]. The unsymmetrical structure of diplopuupehenone comprises puupehenone and puupehenol segments. Bioactivity assays of these compounds unveiled the cytotoxic activity of dipuupehedione (**222**) against KB cells (ED_50_ = 3 μg/mL) [131] and the antioxidant properties of diplopuupehenone (**223**). Compound **223** exhibited the greatest potency in scavenging the 2,2-diphenyl-1-picrylhydrazyl (DPPH) free radical with an IC_50_ value of 8 μM, in comparison with that of puupehenone (**185**, IC_50_ 32 μM), bispuupehenone (**221**), IC_50_ 120 μM) and the standard antioxidant Trolox (IC_50_ 16 μM) [131].

**Figure 16:**
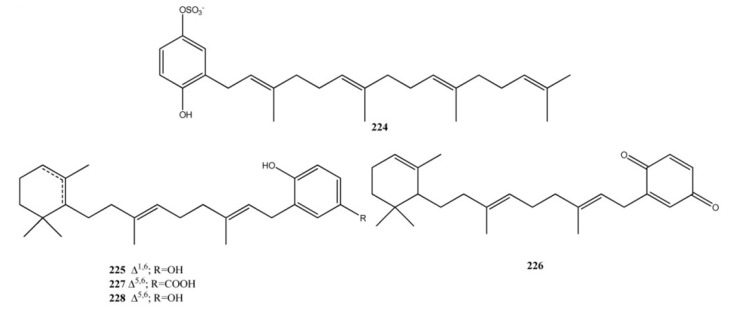
Diterpene benzo(hydro)quinones with linear or monocyclic terpene moieties.

The meroterpene sulfate fascioquinol E (**224**), isolated from a *Fasciospongia* sp., (Figure 16) is a rare example of diterpene benzo(hydro)quinone with a linear terpene moiety [156]. Fascioquinol E is an inhibitor of protein tyrosine phosphatase CpsB, which unexpectedly inhibited the growth of Gram-positive pathogens. CpsB is a member of the polymerase and histidinol phosphate phosphatase (PHP) domain family. Another member of this family found in a variety of Gram-positive pathogens is DNA polymerase PolC and this competes away fascioquinol E inhibition of CpsB phosphatase activity. It was showed that fascioquinol E not only inhibits the phosphatase activity of CpsB, but also ability of PolCPHP to catalyse the hydrolysis of pNP-TMP. This suggests that PolC may be the essential target of fascioquinol E, and that the PHP domain may represent an as yet untapped target for the development of novel antibiotics [157,158].

Cacospongins B–D (**225**–**227**), from *Cacospongia* sp. and jaspaquinol (**228**) from the sponges *Jaspis splendens* and *Suberea* sp. [159,160] exhibit a monocyclic terpene portion. Compounds **227** and **228** showed significant antimicrobial activity against *S. epidermidis* (MIC = 20 and 5.0 μg/mL, respectively) though weaker in comparison with vancomycin [161,162] (MIC = 0.625 μg/mL). Compound **228** showed also to be active as human 15-lipoxygenase and 12-lipoxygenase inhibitor with IC_50_ = 0.3 ± 0.1 μM and IC_50_ = 4.5 ± 1.0 μM, respectively [163].

**Figure 17:**
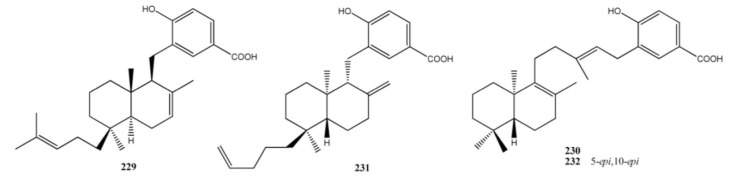
Diterpene benzo(hydro)quinones with bicyclic terpene moieties.

From extracts of two different sponges, *Jaspis splendens* (order Choristida, family Jaspidae) and *Suberea* sp. (order Verongida, family Aplysinellidae), collected in Papua New Guinea, mixed biogenesis bicyclic diterpene-benzenoid compounds, (−)-jaspic (**229**) and (−)-subersic (**230**) acids have been isolated (Figure 17) [163]. Both compounds **229** and **230** were active as human 15-lipoxygenase (15-hLO) inhibitors (IC_50_ values between 0.3 and 15.0 μM) and **229** was also shown to inhibit 12-lipoxygenase (12-hLO) (IC_50_ = 0.7 ± 0.05). (+)-Isojaspic acid (**231**) has been isolated from *Cacospongia* sp. and it has shown a significant antimicrobial activity against *S. epidermidis* (MIC = 2.5 μg/mL) [161]. Interestingly, the enantiomer of the (−)-subersic acid (**230**), the (+)-subersic acid (**232**), isolated from *Acanthodendrilla* sp., inhibited the protein kinase MK2 with IC_50_ of 9.6 μM [164].

**Figure 18:**
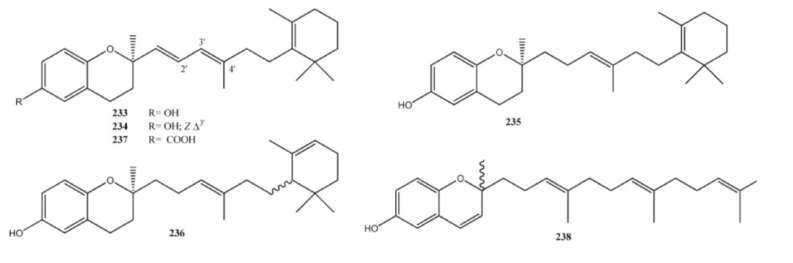
Chromane meroditerpenes.

Chromane meroditerpenes (Figure 18) have been isolated from *Psammocinia* and *Fasciospongia* sp. Bioassay-guided fractionation of the active *Psammocinia* extract led to the isolation of a series of hLO inhibitory chromanes meroditerpenes named chromarols A–E (**233**–**237**) [165]. These compounds were tested in order to determine their comparative inhibition against 15-hLO and 12-hLO. The results of this analysis were quite remarkable, with chromarols A–D (**233**–**236**) exhibiting selective (>25–166-fold) inhibition against 15-hLO versus 12-hLO. Furthermore, **233**–**236** were relatively potent inhibitors of 15-hLO, exhibiting IC_50_ values ranging from 0.6 ± 0.1 to 4.0 ± 0.5 μM. In stark contrast, the 6-carboxychromane derivative, chromarol E (**237**), exhibited no selectivity for either isozyme. Instead, **237** displayed comparable potency against both 15-hLO and 12-hLO (IC_50_ = 3.3 ± 0.4 and 1.2 ± 0.1 μM, respectively). A new metabolite, fascioquinol F (**238**), with a structure comparable to those of chromarols, has been isolated from *Fasciospongia* sp. [156].

**Figure 19:**
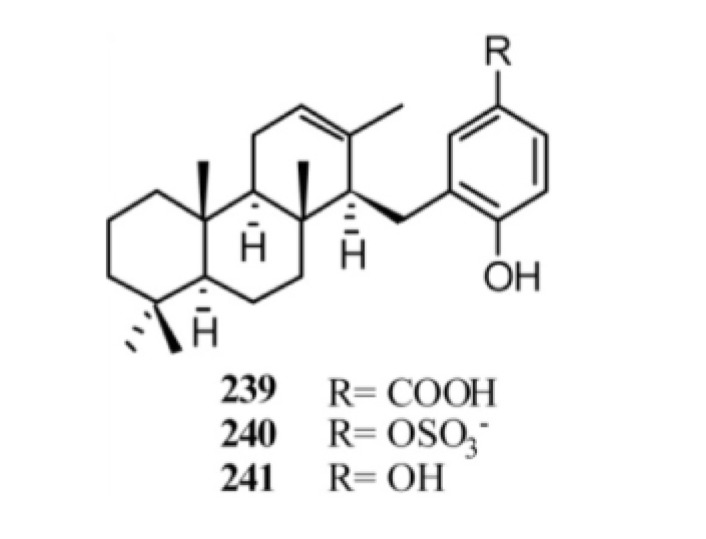
Diterpene benzo(hydro)quinones with tricyclic terpene moieties.

The screen of marine sponges *Acanthodendrilla* sp. and *Fasciospongia* sp. led to isolation of diterpene benzo(hydro)quinones with tricyclic terpene moieties (Figure 19). The extract of *Acanthodendrilla* sp., collected in Indonesia, led to the isolation of (+)-makassaric acid (**239**) which also is an inhibitor of the protein kinase MK2 with an IC_50_’s value of 20 μM [164]. Chemical investigation of a southern Australian deep-water marine sponge, *Fasciospongia* sp., returned the new meroterpene sulfate fascioquinol A (**240**) together with its desulfated analog, fascioquinol B (**241**). Both compounds **240** and **241** displayed a promising Gram-positive selective antibacterial activity towards *Staphylococcus aureus* (IC_50_ 0.9–2.5 μM) and *Bacillus subtilis* (IC_50_ 0.3–7.0 μM) [156].

**Figure 20:**
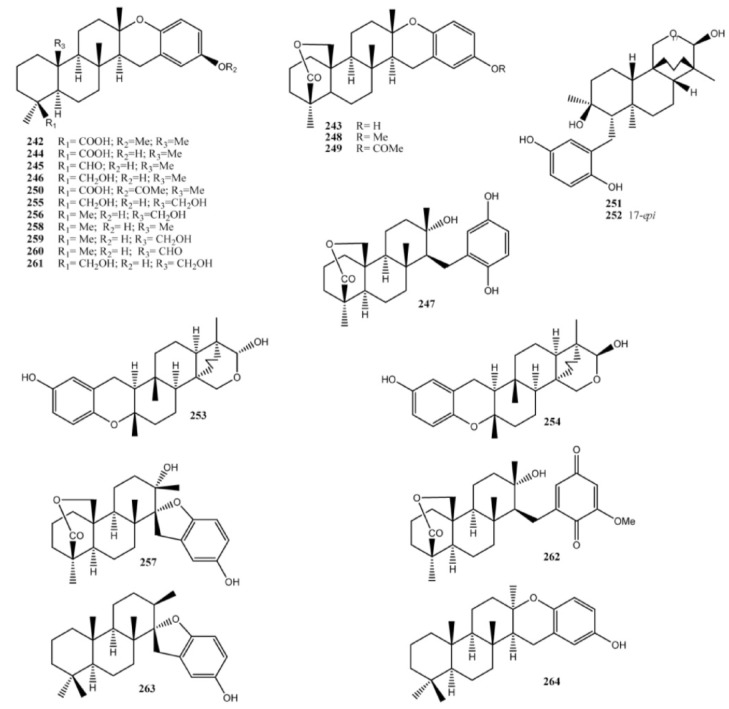
Strongylophorines and fascioquinols C and D.

Meroditerpenoids with more complex structures featuring further oxygen spiranic or dihydropyranic rings, are strongylophorines (**242**–**262**) (Figure 20), first reported from the sponge *Strongylophora durissima* collected in Papua New Guinea [166] and in the Philippines [167]. Strongylophorines exhibited several biological activities including antimicrobic and insecticidal activities, lethal toxicity to brine shrimp, and inhibition of the maturation of starfish oocytes [168,169,170,171,172]. Strongylophorines-8 (**247**) and -26 (**262**) were found to have potent antiinvasive activity using MDA-MB-231 breast cancer cells [173], whereas the strongylophorines 2 (**243**), 3 (**244**), and 8 (**247**) inhibited the HIF-1-dependent luciferase expression in U251-HRE cells with EC_50_ values much lower than their cytotoxic concentrations [174]. In addition, further studies have shown that strongylophorine-8 (**247**) activated the Nrf2/ARE pathway and protected neuronal cells against oxidative stress, representing the first example of a neuroprotective pro-electrophilic compound from marine organisms [175]. Related to strongylophorines are fascioquinols C (**263**) and D (**264**), isolated from *Fasciospongia* sp. [156].

**Figure 21:**
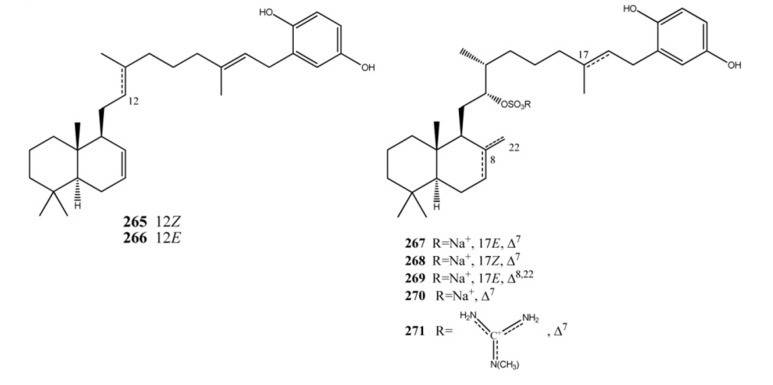
Sestermeroterpenes: coscinoquinols, coscinosulfates, and halisulfates.

Coscinoquinol (**265**), its isomer **266**, and the related sulfate-containing compounds halisulfates and coscinosulfates (**267**–**271**) (Figure 21) are examples of sestermeroterpenes [176,177,178,179]. Halisulfates and coscinoquinols have been reported to possess diverse bioactivities: cytotoxicity, antimicrobial activity, and inhibitory activities against isocitrate lyase, phospholipase A2, serine protease, phosphatase, and PMA-induced inflammation [177,178,179,180,181,182,183]. Significant structure-activity relationships have been evidenced in this compounds family. Compounds **265**, **267** and **269** were active (MIC 1.56–25 μg/mL) against Gram-positive (*Bacillus subtilis*, *Micrococcus luteus*, and *Staphylococcus aureus*) and/or -negative (*Proteus vulgaris* and *Salmonella typhimurium*) bacteria. Interestingly, despite the significant inhibition shown by halisulfate 1 (**267**), its isomer **268** displayed no antibacterial activity at all. This same phenomenon was observed between coscinoquinol (**265**) and its isomer (**266**). Coscinoquinols (**265** and **266**) were also cytotoxic against the K562 cell line, showing more potent inhibition than doxorubicin, while the halisulfates with hydroquinone moiety (**267**–**269**) were inactive. In enzyme-based assays, compounds **267**–**269**, exhibited significant inhibition of sortase A, a key enzyme for the cell adhesion of Gram-positive bacteria; this activity was related to the presence of both hydroquinone and sulfate functionalities. In an assay against *Candida albicans*-derived isocitrate lyase (ICL), an enzyme of the glyoxylate cycle in microorganisms, the coscinoquinols were inactive, while all other sulfate-containing compounds displayed moderate to significant inhibition. A similar trend was observed for the Na+/K+-ATPase inhibitory assay, in which more potent activities were found for the sulfate-containing compounds (**267**–**268**) than the coscinoquinols (**265** and **266**) [176]. Coscinosulfate (**270**) displayed significant inhibitory activity towards CDC25A phosphatase with an IC_50_ of 3 μM, while its analog (**271**) was less active (IC_50_ value of 18 μM) [184]. Both compounds **270** and **271** showed also an antimicrobial activity towards *S. aureus* with inhibition zones, respectively: 12 mm (50 μg/disk) and 10 mm (100 μg/disk) [179].

**Figure 22:**
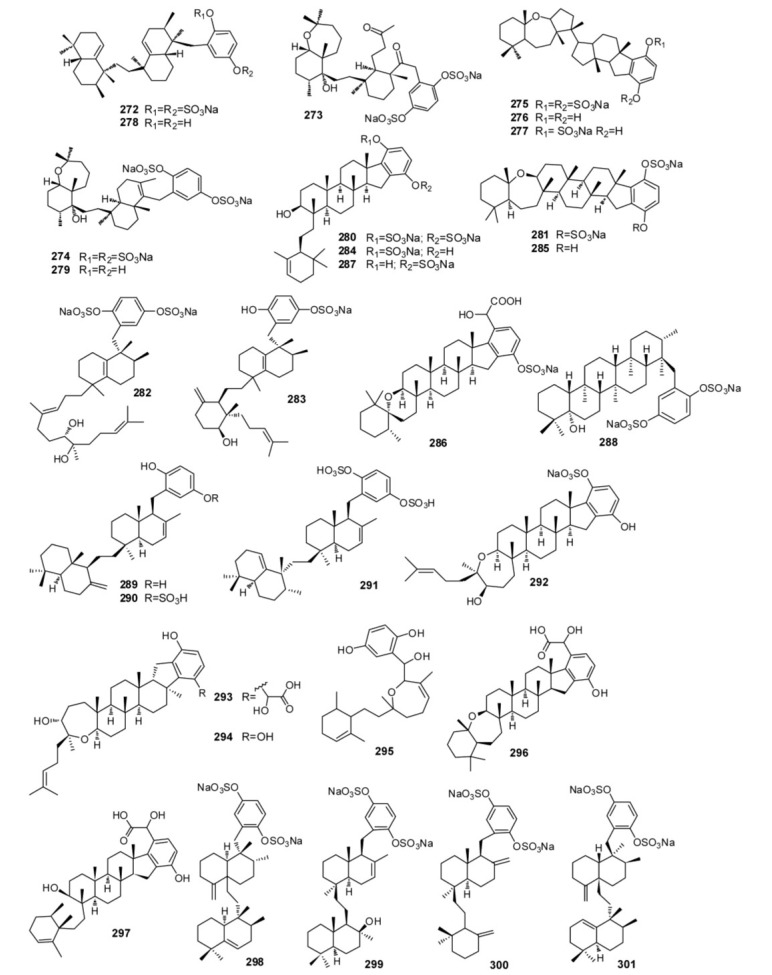
Sulfated triterpene hydroquinones.

Examples of hexaprenoid hydroquinones are reported in Figure 22. The sulfated compounds toxiusol (**272**), shaagrockol B (**273**), shaagrockol C (**274**), toxicol A (**275**), toxicol B (**276**), the p-hydroquinone derivative of compound **275**, and toxicol C (**277**), isolated from *Toxiclona toxius* [185,186], are of interest not only because of their structures but also because of their biological properties, which includes an important activity against human immunodeficiency virus type 1 (HIV-1) reverse transcriptase (RT). The hydrolysis of compounds **272** and **274** yielded the corresponding hydroquinones designated as compounds **278** and **279**, and further oxidation of compound **276** afforded the corresponding p-quinone derivative. Compounds **272**–**276**, **278** and **279** exhibited inhibitory activity of both DNA polymerizing functions of HIV-1 RT but failed to inhibit the RT-associated ribonuclease H activity [74]. The sponges of the genus *Adocia* (order Haplosclerida, family Chalinidae), collected in different places, also are the source of biologically active hexaprenoid hydroquinone sulfates (Figure 22) [187,188,189,190,191]. From the crude extract of *Haliclona* (aka *Adocia*) sp., collected at Palau, it has been possible to isolate adociasulfates 1–6 (**280**–**285**) [187], which contain mono- or di-sulfated hydroquinones, and adociasulfate 10 (**286**) (Figure 22) [188], in which there is an unusual glycolic acid residue in place of one of the sulfates groups. These compounds are inhibitor of the kinesin family of microtubule proteins, a group of proteins that transport cargo along the microtubules within the cells [192,193]. Adociasulfate 10 (**286**) had an IC_50_ of 7 μM, which is almost identical to the activity of adociasulfate 2 (**281**), IC_50_ of 6 μM. It is interesting to note that substitution of one sulfate group by the glycolic acid moiety of adociasulfate 10 (**286**) does not significantly reduce its inhibition of kinesin motors. Independently, adociasulfates 1 (**280**), 7 (**287**), and 8 (**288**), isolated from an unidentified species of *Adocia* collected in Northern Queensland, were found to have H^+^-ATPase protein pump activity [189]. A further investigation of a Palauan specimen of *Haliclona* led to the isolation of three new merotriterpenoids, adociaquinol (**289**), adociasulfates 11 (**290**) and 12 (**291**) [190], whereas an investigation of a sample of *Adocia aculeata* collected from Cormorant Pass, North Great Barrier Reef, led to the isolation of a new triterpene hydroquinone sulfate, adociasulfate 9 (**292**) [191]. Two new terpene-ketides, haliclotriol A (**293**) and haliclotriol B (**294**), have been isolated from two Indo-Pacific members of *Aciona* genus [194]. The major structure of haliclotriols A (**293**) and B (**294**) is derived from the cyclization of a hexaprenoid precursor. The biological activity properties of the triterpenes were investigated: a weak antimicrobial activity was observed for haliclotriol B (**294**) at 1 mg disk against *B. subtilis* and *S. aureus*, respectively.

Halioxepine (**295**), from an Indonesian sample of *Haliclona* sp. [195] showed moderate cytotoxicity against NBT-T2 cells with IC_50_ 4.8 μg/mL and it also exhibited antioxidant activity against 1,1-diphenyl-2-picrylhydrazyl (DPPH) with IC_50_ 3.2 μg/mL as well as other hydroquinone-containing meroditerpenes [196]. Halicloic acids A (**296**) and B (**297**), isolated from a sample of *Haliclona* collected in the Philippines [197], are related to the known compounds adociasulphates 2 (**281**) [187] and 10 (**286**) [188] and haliclotriol A (**293**) [194]. Bioassay-guided fractionation of the extract identified halicloic acids A (**296**) and B (**297**) as indoleamine 2,3-dioxygenase inhibitors. Akaterpin (**298**), isolated from the marine sponge *Callyspongia* sp. having disulfated hydroquinone moiety, is an inhibitor of phosphatidylinositol-specific phospholipase C with an IC_50_ value of 0.5 μg/mL. It also inhibits neutral sphingomyelinase weakly with an IC_50_ of 30 μg/mL [198]. Three new disulfated meroterpenoids, ilhabelanol (**299**), ilhabrene (**300**), both with unprecedented meroterpenoid carbon skeletons, and isoakaterpin (**301**), have been isolated from extracts of the same sponge (Figure 22). They are inhibitors of *Leishmania* spp. adenosine phosphoribosyl transferase (APRT), an important component of the purine salvage pathway in the parasites [199].

## 4. Meroterpenes from Soft Corals

**Figure 23:**
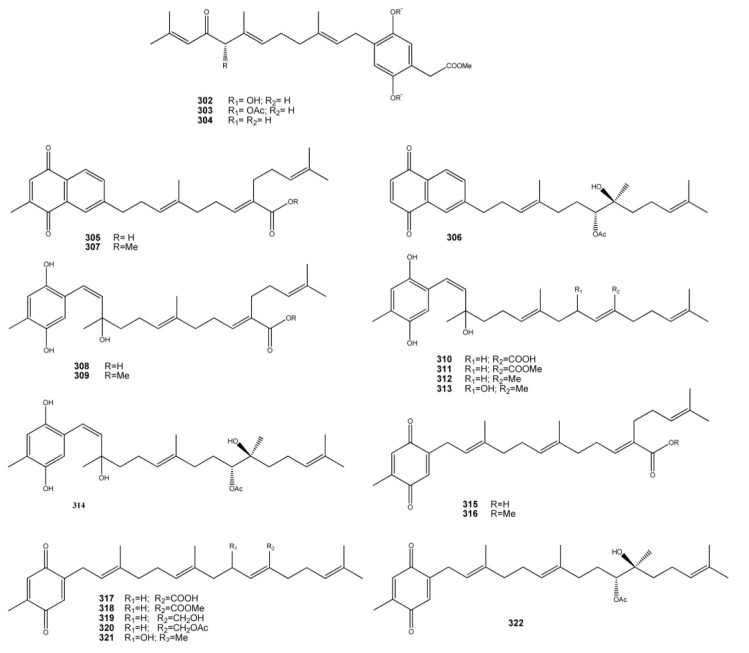
Meroterpenoids from soft corals.

A limited number of meroterpenoids have been isolated from soft corals, the only examples being the geranyl idroquinone derivatives **302**–**304** isolated from the marine octocoral *Alcyonium fauri* [200] and the meroditerpenois **305**–**314** isolated from *Nephthea chabrolii* [201,202,203] (Figure 23).

In a NCI’s CEM-SS cell line screen, designed to detect agents acting at any stage in the HIV virus reproductive cycle, rietone (**302**) showed moderate activity (EC_50_ 1.23 μM and IC_50_ 9.32 μM) [200]. *N. chabrolii* metabolites series include the naphthoquinone derivatives chabrolonaphthoquinone A–C (**305**–**307**), the tetraprenyltoluquinol-related metabolites chabrolohydroxybenzoquinones A–G (**308**–**314**), and the tetraprenyltoluquinones chabrolobenzoquinones A–H (**315**–**322**). Compound **306** exhibited significant cytotoxicity against the growth of the MDA-MB-231 (IC_50_ 4.7 μM) cancer cell line and moderate to weak cytotoxicity against Hep G2 (IC_50_ 12.4 μM) and A549 (IC_50_ 33.9 μM) cancer cell lines, respectively. Also, metabolites **307** and **314** exhibited moderate to weak cytotoxicity toward these cancer cells. Other metabolites either were inactive or exhibit only weak cytotoxicity against the growth of the above three cancer cell lines.

## 5. Conclusive Remarks

**Figure 24:**
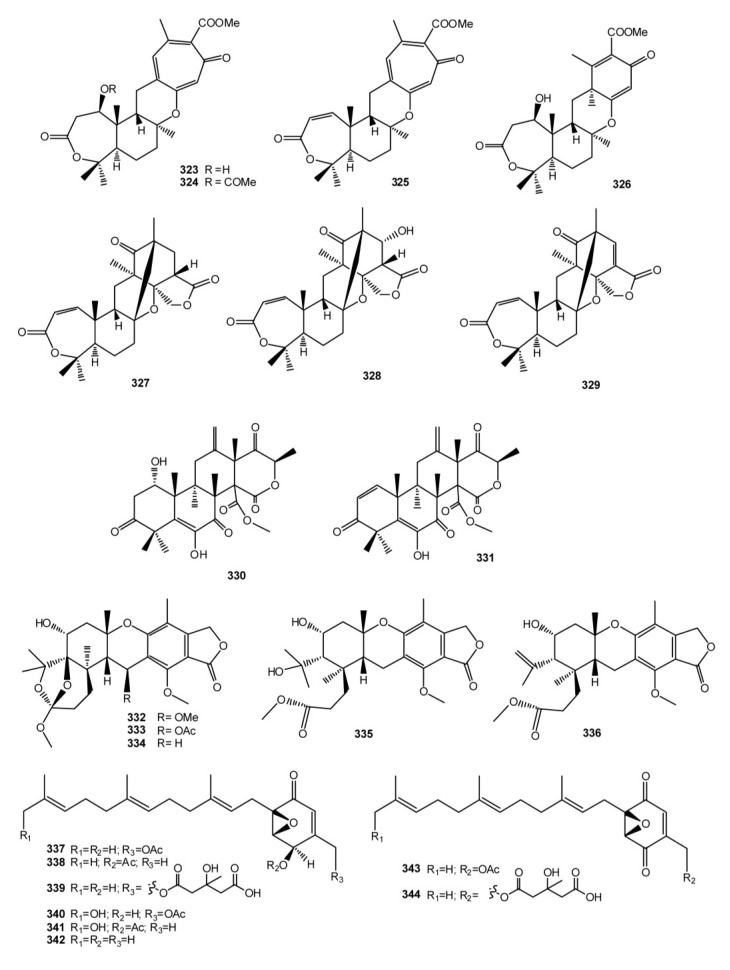
Meroterpenoids from marine invertebrates-associated *Aspergillus* spp.

The great chemical diversity generating in the group of meroterpenes isolated from marine invertebrates and their wide range of biological activities represent a useful tool for development of new therapeutics. But the biomedical potential of these compounds could be greatly enhanced by a comprehensive understanding of their biosynthetic origin combined with the recent progress in molecular biology. The occurrence of different but biosynthetically related meroterpenes in different organisms, in terrestrial sources, and/or in collections of the same organism from distinct geographical locations, strongly supports the possibility of their biosynthesis by associated microorganisms. Significantly, several meroterpenoids have been recently isolated from *Aspergillus* spp. derived from tissues of marine invertebrates. It is known that members of the genus *Aspergillus* can combine polyketide and terpene precursors to produce meroterpenoids, some of whom having important relevance to human health; this is the case of territrem B, produced by *A. terreus*, a potent irreversible inhibitor of acetyl cholinesterase (AChE) and a candidate for drug development for treating Alzheimer’s disease [204]. Examples of meroterpenoids isolated from invertebrate-associated *Aspergillus* spp. are tropolactones A–D (**323**–**326**) isolated from an *Aspergillus* sp. derived from an unidentified sponge [205], insuetolides A–C (**327**–**329**) from *A. insuetus* isolated from the Mediterranean sponge *Psammocinia* sp. [206], terretonins E (**330**) and F (**331**), isolated from *A. insuetus* derived from the Mediterranean sponge *Petrosia ficiformis* [207], austalides M–Q (**332**–**336**) from an *Aspergillus* sp. derived from the sponge *Tethya aurantium* [208], and yanuthones (**337**–**344**) isolated from *A.niger* obtained from tissue homogenates of an *Aplidium* ascidian [209] (Figure 24). Thus, there are grounds to suppose that meroterpenoids isolated from marine invertebrates or, at least, portions of their structure are microbial products, most likely elaborated by *Aspergillus* fungi. If confirmed, this possibility could work to advantage the research on these compounds, both for the exploitation of their huge chemical diversity and for a potential large-scale production of the bioactive molecules. The *Aspergillus* genus of fungi, indeed, has been largely investigated due to its medical and commercial importance. Research on *Aspergillus* has contributed much knowledge about its fundamental cell biology and biochemistry and, foremost, the significance of *Aspergillus* was cause for the sequencing of the genomes of some of the most well-known members of this genus which are now publicly available [210]. Attempts to locate the biosynthetic genes for meroterpenoids production in the genome of some *Aspergillus* spp. have been performed with encouraging results; the biosynthetic pathway for some meroterpenoids (austinol, terretonin) has been proposed [211,212]. Understanding of *Aspergillus* secondary metabolism would greatly profit from the genome sequencing projects; sequence information greatly facilitates the identification of natural product genes, the function of which can be demonstrated by molecular biological and biochemical approaches. When a set of genes involved in the formation of the same secondary metabolite are recognized, a biosynthesis can be proposed. Down the road, such advances should be useful for enhanced production of secondary metabolites of interest and the development of second-generation compounds with improved pharmacodynamic and pharmacokinetic characteristics.Thus, advances in *Aspergillus* secondary metabolite research in the post-genomic era will bring an understanding of meroterpenoids biosynthesis at the genetic level which should facilitate engineering of second generation molecules and increasing production of first generation compounds.
